# Pulmonary blood volume assessment from a standard cardiac rubidium-82 imaging protocol: impact of adenosine-induced hyperemia

**DOI:** 10.1007/s12350-023-03308-1

**Published:** 2023-06-22

**Authors:** Martin Lyngby Lassen, Christina Byrne, Jacob Peter Hartmann, Andreas Kjaer, Ronan M. G. Berg, Philip Hasbak

**Affiliations:** 1 Department of Clinical Physiology, Nuclear Medicine and PET, University Hospital Copenhagen–Rigshospitalet, Copenhagen, Denmark; 2 Cluster for Molecular Imaging, Department of Biomedical Sciences, Faculty of Health and Medical Sciences, University of Copenhagen, Copenhagen, Denmark; 3 Renal, Cardiovascular, and Pulmonary Research, Department of Biomedical Sciences, Faculty of Health and Medical Sciences, University of Copenhagen, Copenhagen, Denmark; 4 Centre for Physical Activity Research, University Hospital Copenhagen–Rigshospitalet, Copenhagen, Denmark; 5 Neurovascular Research Laboratory, Faculty of Life Sciences and Education, University of South Wales, Cardiff, UK

**Keywords:** Transit times, adenosine, pulmonary blood volume, positron emission tomography

## Abstract

**Background:**

This study aimed to assess the feasibility of estimating the pulmonary blood volume noninvasively using standard Rubidium-82 myocardial perfusion imaging (MPI) and characterize the changes during adenosine-induced hyperemia.

**Methods:**

This study comprised 33 healthy volunteers (15 female, median age = 23 years), of which 25 underwent serial rest/adenosine stress Rubidium-82 MPI sessions. Mean bolus transit times (MBTT) were obtained by calculating the time delay from the Rubidium-82 bolus arrival in the pulmonary trunk to the arrival in the left myocardial atrium. Using the MBTT, in combination with stroke volume (SV) and heart rate (HR), we estimated pulmonary blood volume (PBV = (SV × HR) × MBTT). We report the empirically measured MBTT, HR, SV, and PBV, all stratified by sex [male (M) vs female (F)] as mean (SD). In addition, we report grouped repeatability measures using the within-subject repeatability coefficient.

**Results:**

Mean bolus transit times was shortened during adenosine stressing with sex-specific differences [(seconds); Rest: Female (F) = 12.4 (1.5), Male (M) = 14.8 (2.8); stress: F = 8.8 (1.7), M = 11.2 (3.0), all *P* ≤ 0.01]. HR and SV increased during stress MPI, with a concomitant increase in the PBV [mL]; Rest: F = 544 (98), M = 926 (105); Stress: F = 914 (182), M = 1458 (338), all *P* < 0.001. The following test–retest repeatability measures were observed for MBTT (Rest = 17.2%, Stress = 17.9%), HR (Rest = 9.1%, Stress = 7.5%), SV (Rest = 8.9%, Stress = 5.6%), and for PBV measures (Rest = 20.7%, Stress = 19.5%)

**Conclusion:**

Pulmonary blood volume can be extracted by cardiac rubidium-82 MPI with excellent test–retest reliability, both at rest and during adenosine-induced hyperemia.

**Supplementary Information:**

The online version contains supplementary material available at 10.1007/s12350-023-03308-1.

## Introduction

Myocardial positron emission tomography (PET) imaging procedures have grown in numbers in the past decade, driven by technological advances and the more widespread installation of PET systems.^1^ Of myocardial PET examinations, myocardial perfusion imaging (MPI) with Rubidium-82 (^82^Rb) is the most commonly performed test.^1,2^ Routine assessments of ^82^Rb-PET MPI focus on myocardial perfusion and viability and ventricular volumes in resting and during pharmacological stress conditions. Pharmacological stressing triggers coronary vasodilation and is key to unveiling whether reversible myocardial ischemia is present.^3,4^ Apart from these well-established clinical metrics, ^82^Rb-PET may also, in principle, be used to measure the pulmonary blood volume (PBV), that is, the blood volume in the pulmonary circulation.

Physiologically, PBV depends on both cardiac and pulmonary vascular function and is continuously monitored by the cardiopulmonary baroreceptor system. PBV is further critically involved in regulating cardiac output, arterial blood pressure, breathing, and natriuresis.^5,6^ An elevated PBV (central hypervolemia) is considered important for disease presentation and progression both in essential hypertension^7^ and chronic heart failure,^8,9^ while central hypovolaemia may contribute to impaired systemic hemodynamic and blood volume regulation in diseases such as diabetes mellitus,^10^ orthostatic hypotension, neurocardiogenic syncope,^11^ cirrhosis,^12,13^ and chronic obstructive pulmonary disease.^14^ While quantitative PBV estimates may have both diagnostic and prognostic implications in such conditions, PBV has classically been measured by the indicator dilution technique that requires extensive invasive catheterization, as well as repeated blood sampling,^15^ which has halted its wide clinical use. At present, the diagnostic and prognostic value of central hyper- or hypovolaemia thus remain to be established.

In the present study, we evaluated the feasibility of measuring PBV through routine, non-invasive ^82^Rb-PET MPI, an approach that potentially has wide clinical applicability. We provide quantitative PBV estimates, as well as test–retest repeatability assessments in healthy volunteers undergoing ^82^Rb-PET MPI, both at rest and during pharmacological stress with adenosine. Further, this study aimed to evaluate if any sex-specific differences in the PBV exist and if the hemodynamic response affects males and females differently.

## Materials and methods

### Study population

This study comprised 33 young, healthy volunteers (15 female) (median age = 23 years, interquartile range (IQR) = [22; 25]) recruited between September 2016 and March 2017. Inclusion criteria were age > 18 years, no regular consumption of medicine, no known medical condition, and no use of tobacco and euphoric substances (except alcohol) within three months prior to study participation. Exclusion criteria were pregnancy, allergy or intolerance to theophylline or adenosine, any prior medical history of asthma, or inability to adhere to the study protocol. The Scientific Ethics Committee of the Capital Region of Denmark [protocol number H-15009293] and the Danish Data Protection Agency approved this study, and all volunteers provided informed oral and written consent.

### Imaging protocol

#### PET acquisition

The 33 healthy volunteers underwent repeated ^82^Rb rest/ adenosine stress PET/CT MPI as previously described.^16^ A subset consisting of 25 volunteers had successful test–retest assessments, while the remaining eight volunteers had one MPI session discarded because of elevated plasma caffeine concentrations (≥ 1 μg·L^−1^).^16,17^ In brief, the volunteers had injection doses targeting 1100 MBq (30 mCi) ^82^Rb for all MPI sessions obtained on a 128-slice Siemens Biograph mCT PET/CT system. In addition, immediately before the rest scans, the volunteers had a low-dose CT performed for attenuation correction purposes (120 kVp; effective tube current, 26 mA [11 mAs quality reference]) acquired using a free-breathing protocol.^18^ Pharmacological stressing was obtained using adenosine infused at 140 μg·kg^−1^·min^−1^ for 6 minutes, with PET emission acquisition starting 2.5 minutes into the infusion. The volunteers were instructed to abstain from caffeine at least 24 hours before each imaging session.

#### PET reconstruction protocol and data processing

The 6-minute-long PET acquisitions were reconstructed into two image series; one dynamic image series comprised 40 frames (40 × 1 seconds) starting at the beginning of the PET acquisition. The use of 1-s frames was chosen to compromise the image quality in the resulting images (i.e., suppressing image artifacts in the reconstructions) while providing a temporal resolution higher than the usual reconstructions used for myocardial blood flow assessments. An 8-ECG-gated image series were reconstructed as a secondary image series to assess myocardial contractility during optimally adenosine-induced hyperemia employing data from the ^82^Rb bolus arrival to the heart plus 90 seconds until 210 seconds into the PET acquisition.^19^ Both image series were reconstructed using the vendor iterative Ordered Subset Expectation Maximization 3D reconstruction method (2 iterations, 21 subsets), with corrections for time-of-flight and point-spread function. In addition, all data were smoothened using 5-mm Gaussian post-filtering.

#### Assessment of mean bolus transit time and PBV

**Figure 1:**
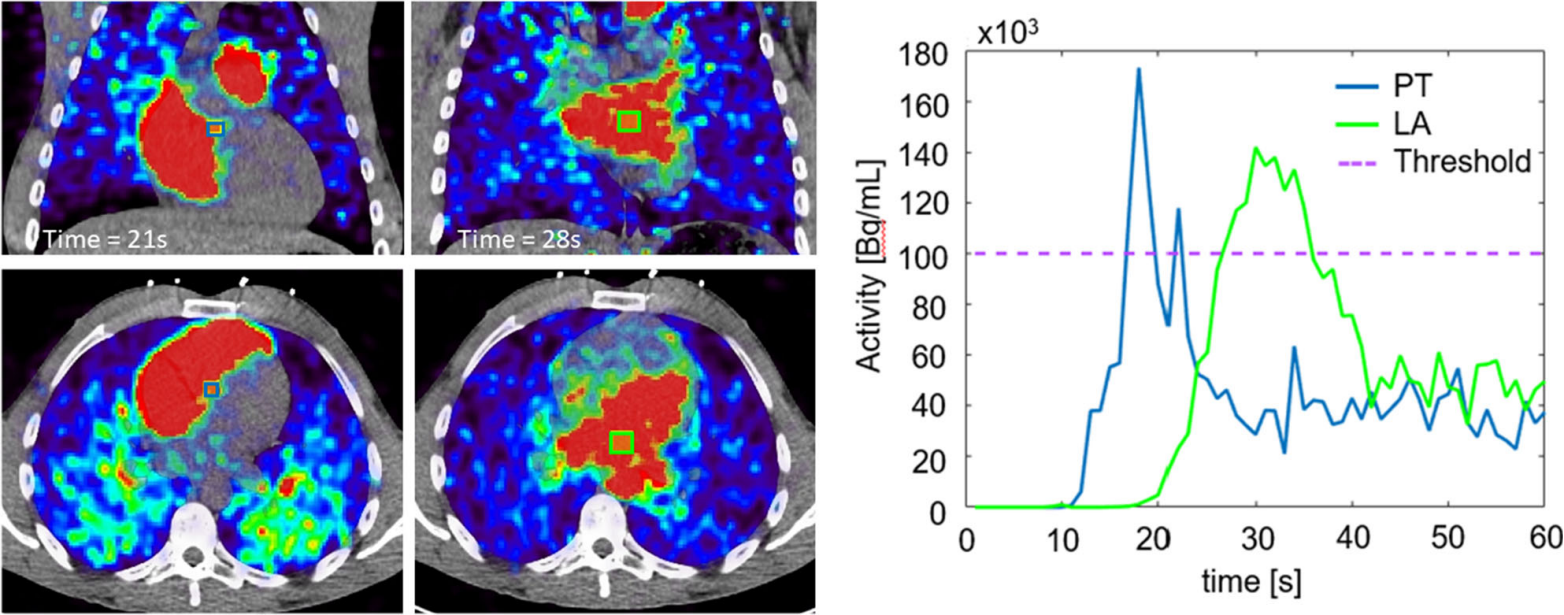
Identification of MBTT. Two volumes of interest were inserted in the base of the pulmonary trunk (10 × 10 × 10 mm^3^, blue box) and left atrium (15 × 15 × 15  mm^3^, green box), respectively. The bolus arrival to either area of interest was defined as a median activity of ≥ 100 kBq or continuous increases in the median activity for five consecutive seconds if the activity did not reach the threshold. *MBTT*, mean bolus transit time; *PT*, pulmonary trunk; *LA* left atrium; *Threshold*, 100-kBq threshold

The mean bolus transit time (MBTT) was determined by inserting cubic volumes of interest into the base of the pulmonary trunk (10 × 10 × 10 mm^3^) and in the left atrium (15 × 15 × 15 mm^3^) (Figure 1). Of note, the discrepancy in the sizes of the volumes of interest was chosen to compensate for the generally reduced size of the pulmonary trunk, which elevates the partial-volume effects in this area compared to the generally homogeneous activity distribution in the left atrium. MBTT was defined as a time delay (seconds) in which the median activity in the volumes of interest exceeded 100 kBq·mL^−1^, thus, defining the arrival of the ^82^Rb bolus. Alternatively, in cases of delayed ^82^Rb dose delivery to the heart (e.g., obstruction of the venous system), the MBTT was defined as the delay of constant activity rise over the course of 5 s in volumes of interest. Of note, the choice of 100-kBq threshold instead for an ordinary peak-to-peak analysis^20^ or area-under-curve analysis was decided upon for two reasons: 1: to compensate for the heterogeneous bolus delivery times provided by the ^82^Rb dose cart used in our center (Bracco diagnostics), which increases throughout the generator lifespan^21^ and 2: to minimize the influence of potential PET system saturation on the assessment of the MBTT; of note PET system saturation is common and may affect as many as 20% of the ^82^Rb MPI studies.^22^

Utilizing the MBTT and the cardiac output (CO) [stroke volume (SV) × heart rate (HR)], the pulmonary blood volume was calculated as (CO × MBTT), such as PBV = MBTT × CO. Of note, the HR was determined as the average value obtained from the listfile (PET raw data) during the first 40 seconds of the acquisition, corresponding to the time period in which the MBTT was determined.

In addition to MBTT and PBV, we report the end-diastolic and end-systolic volumes (EDV and ESV, respectively), the stroke volume (SV), and the HR for all studies. We report all values and their reserves (stress − rest measures) stratified by sex. Of note, the volumetric analyses were extracted from Cedars QPS/QGS (Cedars-Sinai). To compensate for variations in the body mass index in the volunteers, we report both the empirical and body surface area (BSA) normalized PBV values for all volunteers normalized to a standard body surface of 1.73m^2^. The BSA was calculated using the DuBois equation^23^:

1 $$ {\text{BSA}} = 0.007184 \times {\text{Weight}}^{0.425} \times {\text{Height}}^{0.725}. $$

With *Weight* being the body weight in kg and the *Height* in cm.

Of note, for participants with repeat scans, the reported values used in figures and statistical assessments are based on the average of both scans.

### Statistical analysis

Data were quantified in R v. 4.1.2 (The GNU project). For descriptive analyses, we used mean (Standard deviation), range or median, and interquartile range for continuous values. Differences in the metrics were evaluated using one-way analyses of variance (ANOVA). Two-tailed *P*-values < 0.05 were considered statistically significant.

We report the grouped test–retest repeatability coefficient (RC) for eight evaluated metrics (HR, SV, EDV, ESV, CO, MBTT, PBV, and the PBV reserve) using the within-subject coefficient of variation assessment.^24^ In addition, we also provide the smallest real difference (SRD) measures in the respective empirical units.^25^

## Results

**Figure 2:**
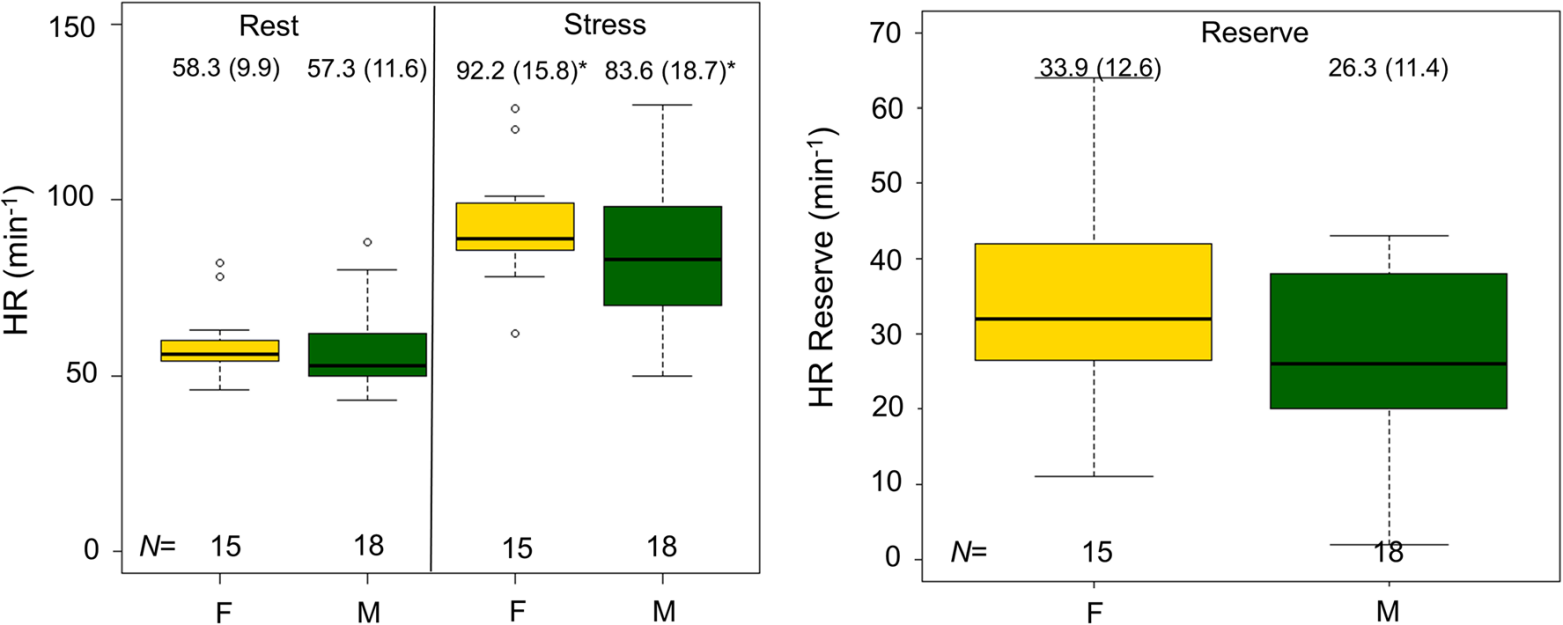
HR and its reserve observed during rest and adenosine stress MPI, stratified by sex. Similar HR and HR reserves were observed for both males and females during both rest and stress MPI. Significant increases in the HR were observed during adenosine stress for both sexes, denoted by *. *HR*, heart rate; *MPI*, myocardial perfusion imaging; *F*, female; M, male

Of the 66 MPI sessions (33 subjects with test–retest assessments), eight were excluded owing to elevated plasma caffeine concentrations (≥ 1 μg·L^−1^), as described in a previous study.^17^ The demographics of the cohort are shown in Table 1. The adenosine-induced hyperemic response increased HR in both males and females, with no sex-specific differences in the HR and its reserve (defined as stress-rest HR) (Figure 2).

**Table 1:** Demographics of the volunteers

	Repeat scans	Full cohort
Number of participants	25	33
Days between test/retest assessment^a^	14 [6; 35]	N/A
Age (years)^a^	22 [22; 23]	22 [22; 23]
Sex, Females, N (%)	11 (44%)	15 (44%)
BMI (kg·m^−2^)^a^	22 [21; 24]	22 [20; 23]
Current smoking, N (%)	9 (36%)	14 (40%)

^a^Median and interquartile range

### Image-derived markers (MBTT, SV, EDV, and ESV)

**Figure 3:**
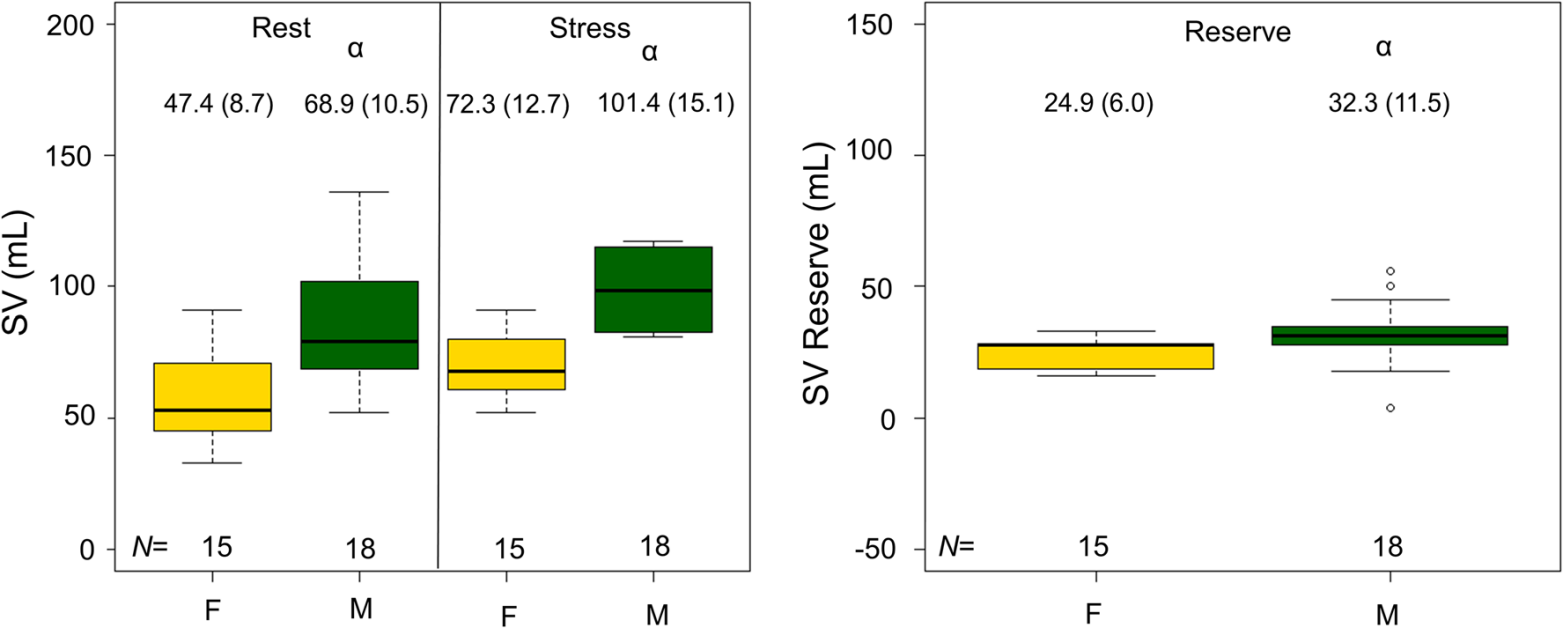
SV observed during rest and adenosine stress MPI, respectively. Significantly elevated SV and SV reserves are reported for males compared to females (marked by α). *marks significant differences between rest and stress MPI. *SV*, stroke volume; *MPI*, myocardial perfusion imaging; *F*, female; *M*, male

**Figure 4:**
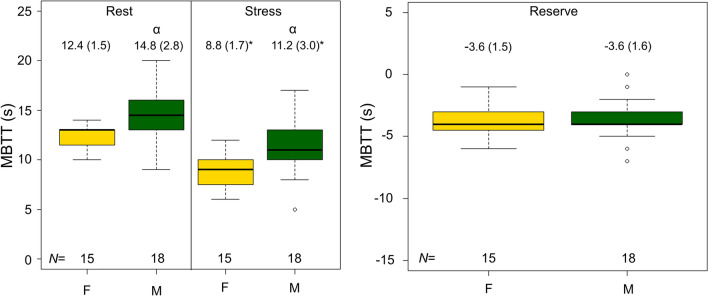
MBTT and its reserve observed for rest and adenosine stress MPI stratified by sex. The MBTT was significantly shortened during adenosine stress MPI (marked by *), with females having shortened MBTT compared to men for both rest and stress MPI (marked by α). No differences were reported MBTT reserve for the males and females. *MBTT*, mean bolus transit time; *F*, female; *M*, male

Stroke volume was observed to elevate significantly during adenosine stressing, with sex-specific differences reported for both rest and stress MPI, as well as the reserve (stress-rest SV) (Figure 3, Table 2). The changes in the SV were driven by slightly elevated end-diastolic volumes observed during stress MPI (Supplementary Figure 1) and slightly reduced end-systolic volumes observed during stress MPI (Supplementary Figure 2), although non-significant. In addition, MBTT shortened during adenosine stress for both sexes, with an average MBTT (combined for both sexes) of 13.7 (2.9) seconds during rest MPI and 10.1 (2.7) seconds (*P* < 0.001) during stress MPI (Figure 4, Table 2). Of note, the males had significantly prolonged MBTT (*P* ≤ 0.01) (Figure 4, Table 2).

**Table 2:** Empirical measurements of EDV, ESV, SV, HR, CO, MBTT, and PBV

	Rest	Stress	Reserve
EDV (mL) (F/M)	75 ± 16/126 ± 22^α^	93 ± 19*/153 ± 26*^,α^	19 ± 6/27 ± 11^α^
ESV (mL) (F/M)	27 ± 8/58 ± 15^α^	21 ± 7/52 ± 14^α^	− 6 ± 3/− 6 ± 7
SV (mL) (F/M)	47 ± 9/69 ± 11^α^	72 ± 13/101 ± 15^α^	25 ± 6/32 ± 12^α^
HR (min^−1^) (F/M)	58 ± 10/57 ± 12	92 ± 16*/84 ± 19*	34 ± 13/26 ± 11
CO (L*min^−1^) (F/M)	2.7 ± 0.6/3.9 ± 0.8^α^	6.6 ± 1.6*/8.4 ± 2.1*^,α^	3.9 ± 1.2/4.5 ± 1.7
MBTT (seconds) (F/M)	12 ± 2/15 ± 3^α^	9 ± 2*/11 ± 3*^,α^	− 4 ± 2/− 4 ± 2
PBV (Empirical) (mL) (F/M)	544 ± 98/926 ± 105^α^	914 ± 182*/1458 ± 338*^,α^	370 ± 137/532 ± 299^α^
PBV (BSA harmonized) (mL) (F/M)	555 ± 79/790 ± 94^α^	946 ± 185*/1259 ± 269*^,α^	391 ± 147/470 ± 262

*EDV*, end-diastolic volume; *ESV*, end-systolic volume; *SV*, stroke volume; *HR*, Heart rate; *CO*, cardiac output; *MBTT*, mean bolus transit time; *PBV*, pulmonary blood volume; *BSA*, body surface area; *F*, female, *M*, male

*Significant changes between rest and stress, while ^α^ denotes sex-specific differences (both shown at stress for the respective sexes). Of note, the measures for participants with repeat scans are reported using the mean of the two scans.

### Hybrid values (CO and PBV)

**Figure 5:**
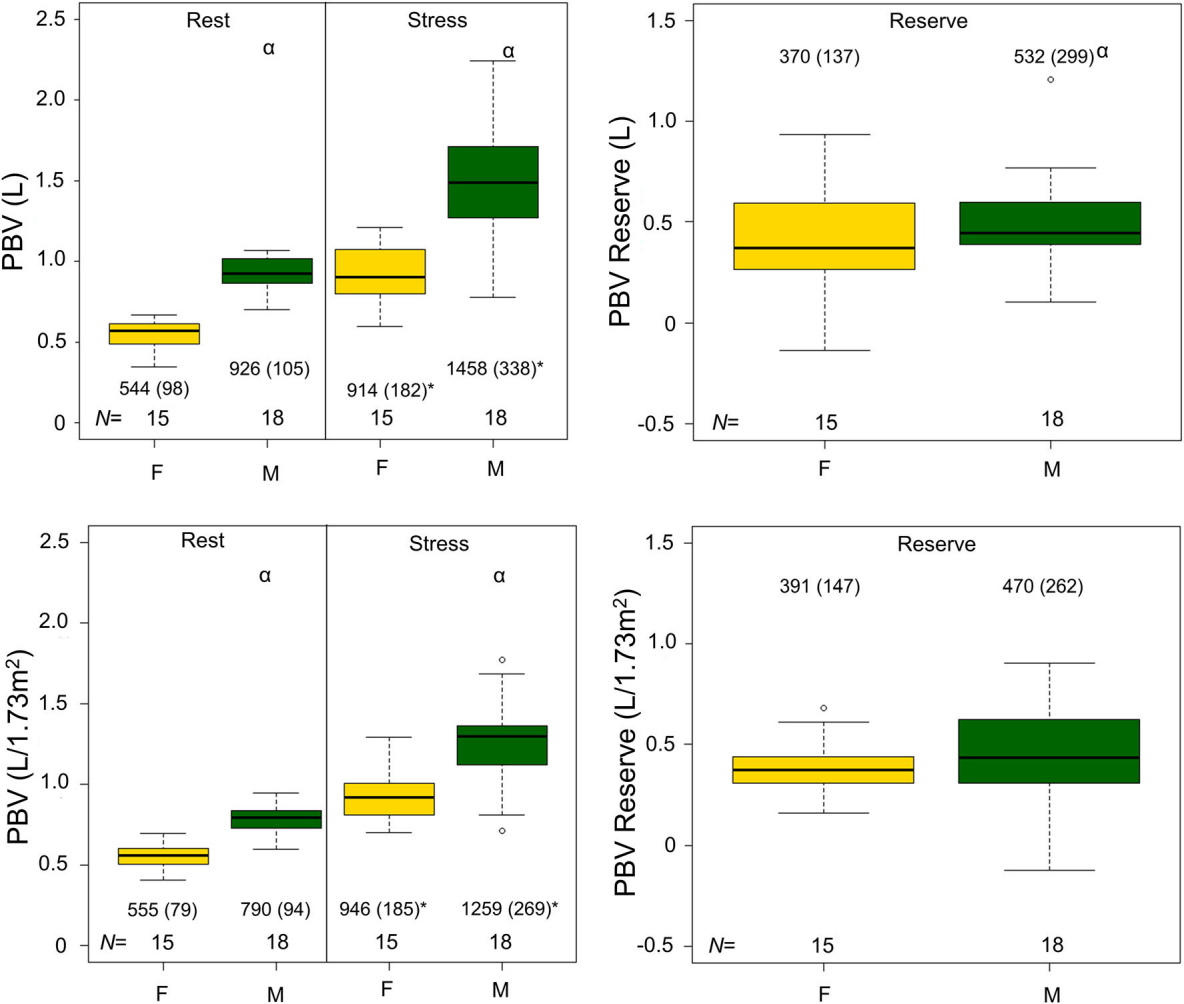
PBV and PBV reserve obtained for rest/adenosine stress MPI, stratified by sex. Upper row shows the empirical measured PBV, while the lower row shows body surface area harmonized PBV measures. PBV and PBV reserve values were significantly elevated for males when data were not harmonized for body surface. Following DuBois correction (body surface area correction), significant differences in the PBV were reported for the two sexes; however, PBV reserve was reported as comparable. Common for both datasets and sexes was a significant increase in the PBV during adenosine stress. * denotes significant differences between rest and adenosine stress MPI, α denotes sex differences. Of note, all PBV values for the respective sexes are given in mL or mL/1.73 m^2^). *PBV*, pulmonary blood volume; *MPI*, myocardial perfusion imaging

The increases in the HR and SV elevated the CO during adenosine stress MPI (Supplementary Figure 3). Adenosine-induced hyperemia elevated the PBV compared to resting condition (grouped PBV measures [mL]: Rest = 752 (217), Stress = 1211 (388), PBV Reserve = 459 (250), all *P* < 0.001, Table 2). Sex-specific differences were reported for rest and stress PBV and its reserve (stress-rest PBV) (Figure 5). Normalizing the data to a standard body surface area (BSA correction) reduced the bias in the PBV reserve between the sexes [grouped BSA normalized PBV (mL/1.73 m^2^): Rest = 683 (147) Stress = 1117 (280), *P* < 0.001; BSA normalized PBV reserve = 434 (218)] (Figure 5, Table 2).

### Repeatability

Grouped (males and females combined) test–retest repeatability measures for all ten empirically measured metrics were acceptable (RC within the range [4.1: 20.7 %]), while the reported reserve values generally performed poorer (RC within the range [22.4: 67.5 %]) (Table 3).

**Table 3:** Test–retest variability of ^82^Rb imaging-derived central hemodynamic parameters (N = 25)

	Rest		Stress		Reserve	
	RC (%)	SRD (units)	RC (%)	SRD (units)	RC (%)	SRD (units)
HR (beats·min^−1^)	9.1	1.3	7.5	1.0	36.8	5.0
EDV (mL)	5.9	0.8	4.1	0.6	30.7	20.4
ESV (mL)	10.8	1.5	8.1	1.1	46.1	6.8
SV (mL)	8.9	1.2	5.6	0.8	43.6	6.1
CO (mL·min^−1^)	13.8	1.9	8.5	1.1	22.4	3.1
MBTT (seconds)	17.2	2.4	17.9	2.5	55.1	11
PBV (mL)	20.7	2.9	19.2	2.6	67.5	9.3
PBV (mL/1.73 m^3^)	20.7	2.9	19.5	2.6	67.2	9.2

The within-subject repeatability coefficient is presented as RC; SRD: Smallest real difference (SRD)

*HR*, heart rate, *EDV*, end-diastolic volume, *ESV*, end-systolic volume, *SV*, stroke volume, *CO*, cardiac output, *MBTT*, mean bolus transit time; *PBV*, pulmonary blood volume

## Discussion

The main finding of the present study was that PBV might be extracted from routine ^82^Rb MPI studies, with excellent repeatability both at rest and during pharmacological stress with adenosine. We found that adenosine-induced hyperemia significantly elevates PBV, with sex-specific differences in the reported volumes. However, BSA normalization harmonizes the PBV reserve across the two sexes.

Our PBV estimates, including sex differences, agree well with those obtained by the indicator dilution technique in healthy supine individuals. In this study, we report grouped rest PBV volumes of 748 (234) mL before BSA normalization, findings which are slightly elevated compared to previous reporting for cardiac MR studies (607 (112) mL).^20^ The observed bias in the PBV values may be expected to be caused by age differences in the cohorts evaluated in our study and the study by Nelsson et al. (median age = 23 years and 61 (8) years, respectively). Further, some of the bias may be attributed to the difference in the definition of MBTT. In our study, we defined the MBTT using a 100-kBq threshold assessment, compared to the other non-standardized MBBT definitions used in in previous studies.^20,26^ Further, the MBTT obtained for ^82^Rb may be affected by first-pass retention in the lungs, which likely will contribute to an extended transit time. In a study by Senthamizhchelvan et al, they report comparable full-width at half maximum for time-activity curves obtained in the myocardial cavities and the lungs, however, with significantly elevated activities observed in the lungs.^27^ The discrepancies in the AUC of the two organs may indicate a first-pass retention of approximately 25%, thus, suggesting an approximately overestimated MBTT of 25% in this study. While being a limitation of ^82^Rb MBTT assessment, it is inherent to the radiotracer; thus, we did not correct this in the current study. Finally, the observed sex differences in PBV are in concordance with the general findings in nuclear cardiology studies, where the rest myocardial blood flow has been reported to be higher in females.^16^

In the present study, adenosine infusion caused a remarkable increase in PBV of approximately 384 mL (in females) and 566 mL (in males), respectively. This has not previously been reported in humans but agrees well with previous findings in dogs undergoing a similar adenosine infusion protocol.^28^ This PBV increase conceivably reflects the combined effects of adenosine on cardiac output and pulmonary vascular tone, as it is known to be a pulmonary vasodilator.^29^ Therefore, the estimation of PBV by ^82^Rb MPI may also help reveal whether sufficient hyperemic response is achieved under pharmacological stressing. The insufficient hyperemic response has been reported to occur frequently in the clinical routine, either through elevated plasma caffeine levels in the blood or poor response to the pharmacological stressing agent.^16^ Of consequence, the inadequate hyperemic response during pharmacological stress may cause false-negative findings and, thus, adversely affect patient care.^30^ A previous study from our center has shown that a selection of physiological, image-derived, and hybrid markers only provide acceptable identification of the adequate hyperemic response.^17^ While not being evaluated in this study, the increase in PBV during adenosine stress may help identify whether sufficient hyperemic response is achieved during stress MPI in patients without heart failure, which may have disease-induced changes in the PBV.

High test–retest repeatability is a critical prerequisite for any new measure to be translated into clinical use. In this study, we evaluated the test–retest repeatability measures of all components included in the PBV assessments. Common for the test–retest repeatability measures was excellent repeatability for most values, with interscan variations < 20% (Table 1). The poorest repeatability measures were observed for the MBTT and PBV measures, with test–retest variations of up to 19% for the empirical measures, while the repeatability measures for the reserves not unexpectedly varied more and up to 67%. While elevated test–retest variation was reported for the MBTT and PBV values, the variations were comparable to test–retest variations observed for assessments of myocardial blood flow and their reserves.^31,32^ Therefore, both MBTT and PBV are of clinical standard and, thus, permittable to be tested in cohorts evaluating patients in future studies.

Although this study focused on assessing the PBV using ^82^Rb MPI, this technique is not limited to nuclear cardiology but may be applicable to other imaging modalities where the MBTT can be estimated. Such studies may include cardiac magnetic resonance imaging, cardiac computed tomography, and ultrasound, as well as other dynamic studies of the heart in nuclear medicine with tracer injection during the acquisition while the heart is in the field of view.

### Study limitations

This study has several notable limitations. First, this study focused on volunteers without any pre-existing conditions and comprised only 33 subjects (of which 25 had repeated measures). Despite the few participants, we report increases in PBV during adenosine stressing of the volunteers with intermediate to high test–retest variability measures. While the cohort of healthy volunteers may be a limitation, we consider this a strength for this study as it permits reliable assessments of the patients without any co-morbidities affecting the changes in PBV during hyperemic response or the test–retest repeatability measures. Several studies evaluating various systemic diseases and their impact on PBV have been initiated to evaluate whether systemic diseases affect PBV. Another limitation of the study is PBV’s reliance on a “geometric” estimation of cardiac output as SV × HR since it could potentially overestimate cardiac output in older patients at greater risk of valvular disease. Further, the bias between the PBV reported in the current study and previous studies may be attributed to bias in the SV assessments obtained in various imaging modalities with different inherent imaging influences (partial-volume effects, positron range, filtering, etc.). Unfortunately, it was not possible to validate the SV against other imaging modalities in this study, and thus, it is a limitation of the validation of the proposed technique. Relating to the method, it is a limitation that the proposed method is more prone to noise artifacts in the MBTT assessment given the threshold-based assessment. Ideally, more reliable MBTT assessments may be obtained from area-under-curve assessments, known from the injection dye technique. Unfortunately, the variable ^82^Rb injection volumes and times render this technique infeasible in routinely acquired studies. Further, the potential first-pass retention of ^82^Rb in the lungs may delay the bolus transit, thus, increasing the MBTT. The retention of the radiotracer is subject to follow up studies evaluating the limitations of the proposed technique. Finally, the study also has the limitation that no invasive measured reference value for the cohort exists and that PBV values can only be compared to other non-invasive methods, such as MRI studies where other methodologies for assessing the bolus transit time are employed. Unfortunately, we did not have ethical permission to evaluate the PBV invasively in this cohort of young, healthy volunteers.

## New knowledge gained

In this study, it has been demonstrated that both the pulmonary blood volumes may be estimated from routinely acquired ^82^Rb MPI acquisitions and that adenosine-induced hyperemia increases PBV in a cohort of healthy volunteers. Further, the PBV obtained from ^82^Rb scans in this study is comparable to previous reports evaluating cardiac MRI studies. Finally, it has been shown that PBV is a reproducible measure, with test–retest variations comparable to those reported from ^82^Rb myocardial blood flow and flow reserve assessments.

## Conclusion

In this proof-of-concept study, we have shown that non-invasive measurements of PBV are feasible using routine MPI PET, with repeatability comparable to other measures used in nuclear cardiology routine. Furthermore, we report that adenosine-induced hyperemia increases PBV in a cohort of healthy volunteers, which has not previously been reported in humans. Finally, we report that some sex-specific differences do exist. However, PBV elevate harmoniously in males and females when the data are normalized for differences in the body surface area.

### Supplementary Information

Below is the link to the electronic supplementary material.

Supplementary file1 (DOCX 349 KB)

Supplementary file2 (PPTX 326 KB)

## Abbreviations

^82^Rb: Rubidium-82
CO: Cardiac output
EDV: End-diastolic volume
ESV: End-systolic volume
HR: Heart rate
MBTT: Mean bolus transit time
MPI: Myocardial perfusion imaging
PET/CT: Positron emission tomography/computed tomography
PBV: Pulmonary blood volume
SV: Stroke volume
