# Supplementary material for: Pulmonary blood volume assessment from a standard cardiac rubidium-82 imaging protocol: impact of adenosine-induced hyperemia
Source: J Nucl Cardiol. 2023 Jun 22;30(6):2504–13. doi: 10.1007/s12350-023-03308-1 (PMC10682170; doi:10.1007/s12350-023-03308-1)
Supplement: Supplementary file 1 — Supplementary file1 (DOCX 349 KB) [file 12350_2023_3308_MOESM1_ESM.docx]

Supplementary material to:

Pulmonary blood volumes may be assessed from standard cardiac Rubidium-82 imaging protocols: Impact of adenosine-induced hyperemia

Martin Lyngby Lassen, PhD^1,2^, Christina Byrne, MD, PhD^1,2^, Jacob Peter Hartmann, MD^1,3,4^, Andreas Kjaer, MD, PhD, DMSc^1,2^, Ronan M. G. Berg MD, DMSc^1,3-5^, Philip Hasbak, MD, DMSc^1^

^1^Department of Clinical Physiology and Nuclear Medicine, University Hospital Copenhagen – Rigshospitalet, Copenhagen, Denmark.

^2^Cluster for Molecular Imaging, Department of Biomedical Sciences, Faculty of Health and Medical Sciences, University of Copenhagen, Denmark

^3^Renal, Cardiovascular, and Pulmonary Research, Department of Biomedical Sciences, Faculty of Health and Medical Sciences, University of Copenhagen, Denmark.

^4^Centre for Physical Activity Research, University Hospital Copenhagen – Rigshospitalet, Copenhagen, Denmark.

^5^Neurovascular Research Laboratory, Faculty of Life Sciences and Education, University of South Wales, UK

Corresponding Author:

Martin Lyngby Lassen, PhD, Department of Clinical Physiology, Nuclear Medicine and PET and Cluster for Molecular Imaging, section 4011, Rigshospitalet and University of Copenhagen, Blegdamsvej 9, 2100 Copenhagen, Denmark

Tel: +45 35453520 Fax: +45 35454015

Email: martin.lyngby.lassen@regionh.dk

Short title: ^82^Rb-PET-based CBV

**Supplementary Figure 1.** **EDV measures stratified by sex**. The EDV was elevated significantly during adenosine stress compared to rest MPI for males and females (denoted by * at the stress scans). Further, significant differences between males and females were observed for the empirically measured values and their reserves (characterized by α at the male box plots); of note, all p<0.05.


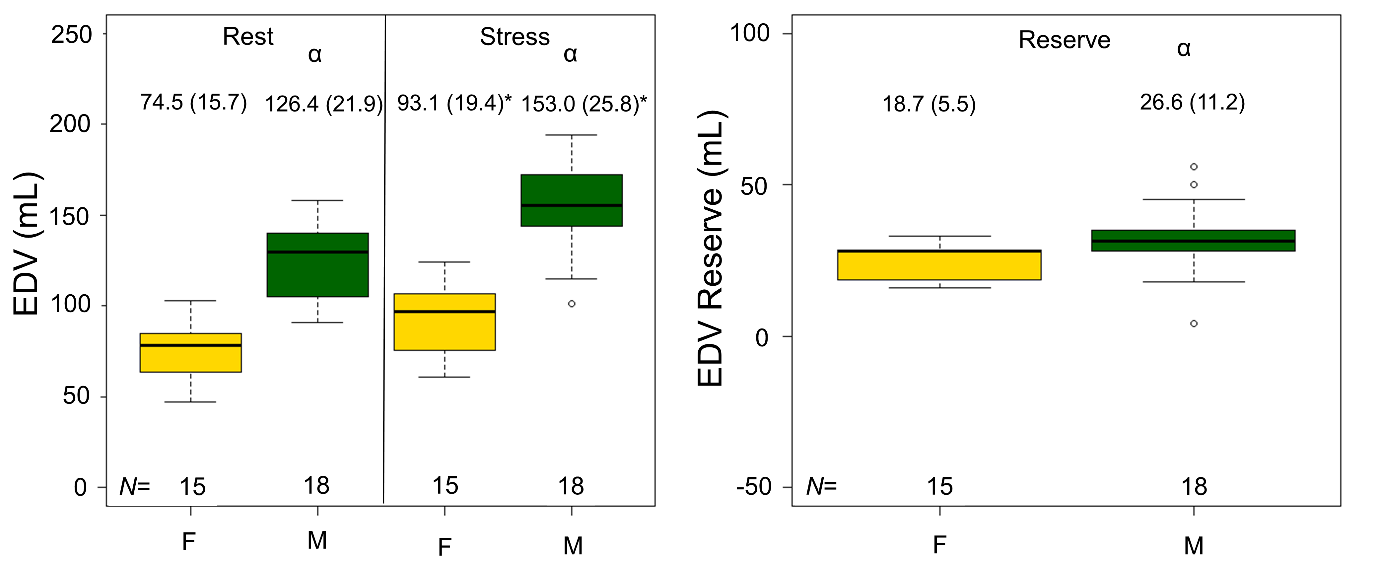


EDV = End diastolic volume, F = female, M = male, MPI = Myocardial Perfusion Imaging.

**Supplementary Figure 2. ESV measures stratified by sex**. No differences were observed in the ESV between rest and stress MPI. Sex-specific differences in the ESV were reported for both rest and stress MPI (denoted by α above the Male boxes), while no differences were reported in the ESV reserve. Of note, both α had p<0.05.


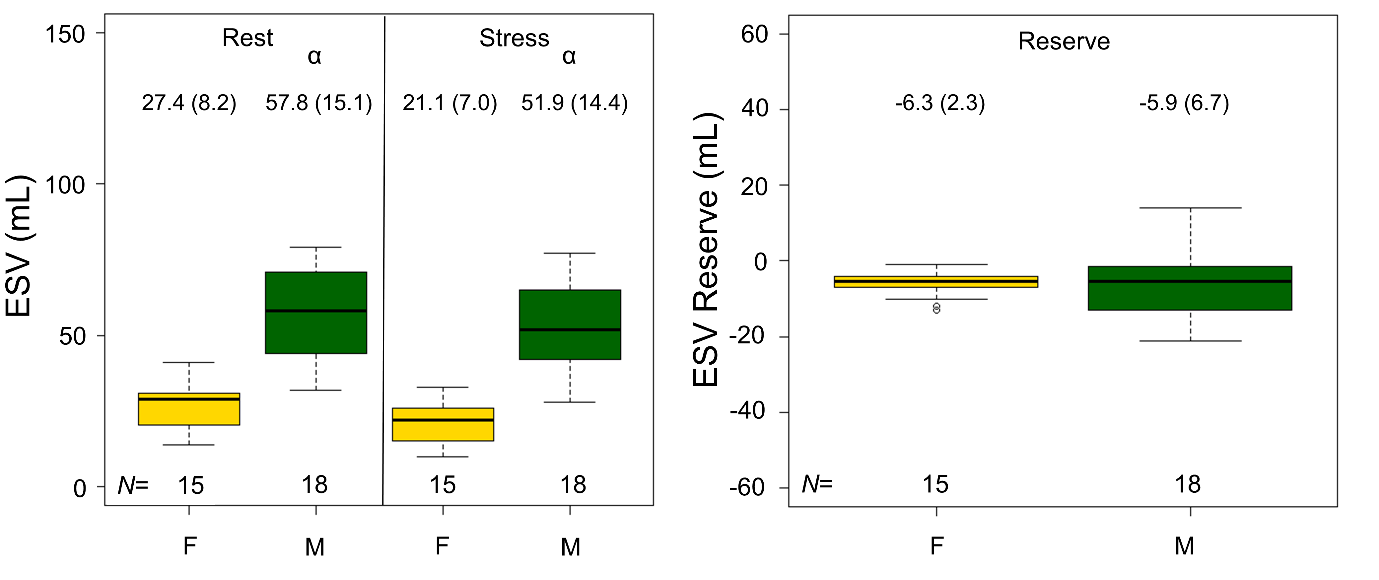


ESV = End systolic volume, F = female, M = male.

**Supplementary Figure 3. CO measures stratified by sex**. Significant increases in the CO were observed during adenosine stress compared to rest MPI for both males and females (denoted by * in the stress box plots). Males were identified to have increased CO measures compared to females (denoted by α above the male box plots), while no differences were observed for the reserves between the two sexes.


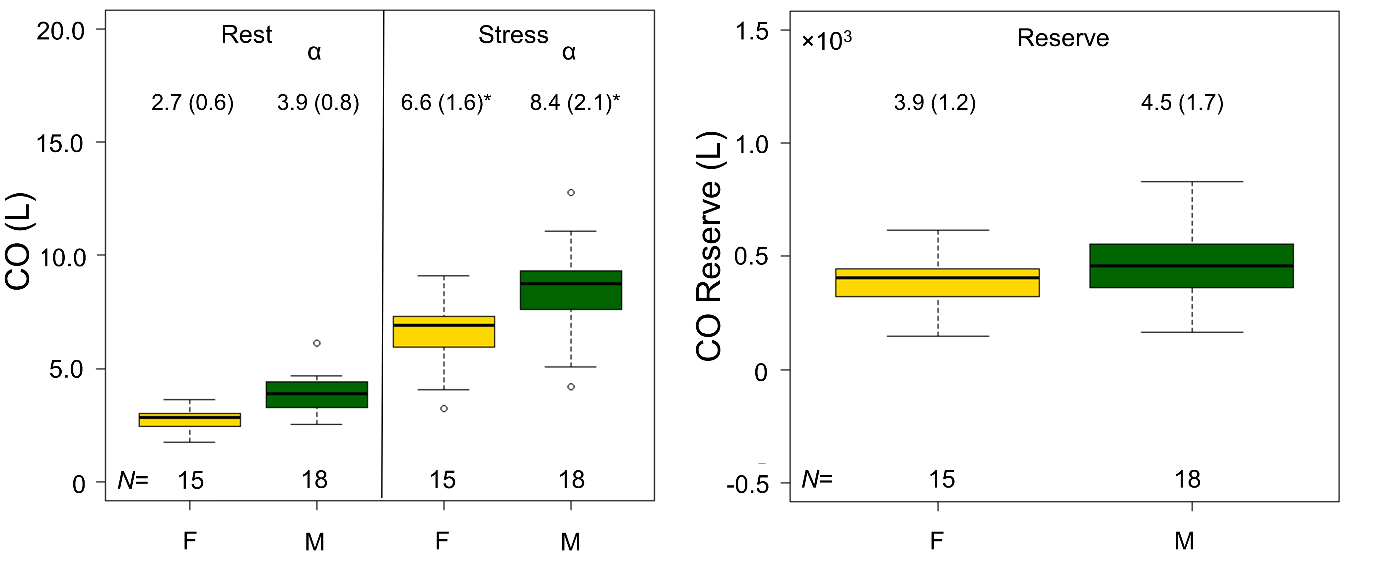


CO = Cardiac output, F = female, M = male.
