# Supplementary material for: Pulmonary blood volume assessment from a standard cardiac rubidium-82 imaging protocol: impact of adenosine-induced hyperemia
Source: J Nucl Cardiol. 2023 Jun 22;30(6):2504–13. doi: 10.1007/s12350-023-03308-1 (PMC10682170; doi:10.1007/s12350-023-03308-1)
Supplement: Supplementary file 2 — Supplementary file2 (PPTX 326 KB) [file 12350_2023_3308_MOESM2_ESM.pptx]

## Slide 1
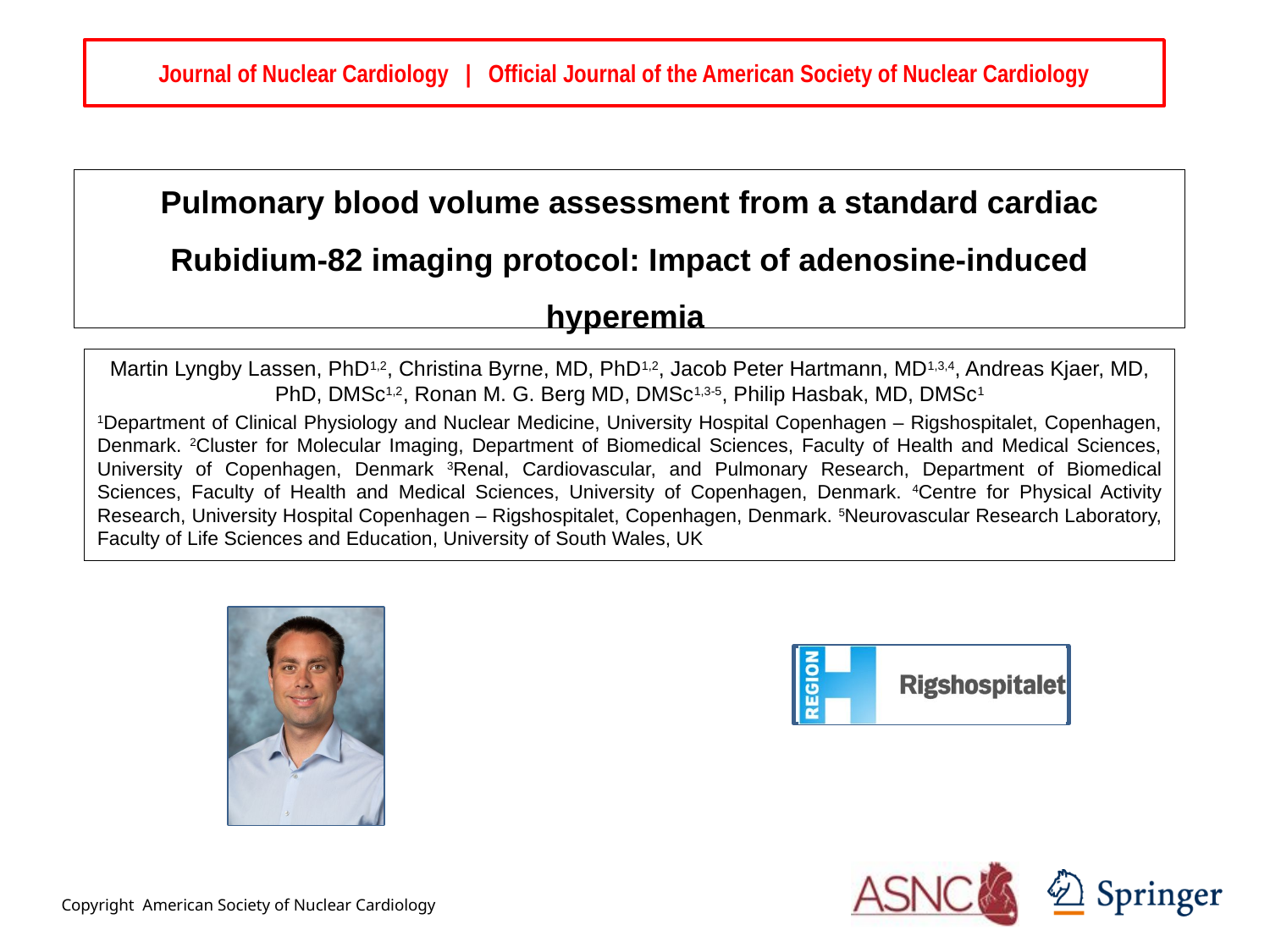

Journal of Nuclear Cardiology | Official Journal of the American Society of Nuclear Cardiology
# Pulmonary blood volume assessment from a standard cardiac Rubidium-82 imaging protocol: Impact of adenosine-induced hyperemia
Martin Lyngby Lassen, PhD1,2, Christina Byrne, MD, PhD1,2, Jacob Peter Hartmann, MD1,3,4, Andreas Kjaer, MD, PhD, DMSc1,2, Ronan M. G. Berg MD, DMSc1,3-5, Philip Hasbak, MD, DMSc1
1Department of Clinical Physiology and Nuclear Medicine, University Hospital Copenhagen – Rigshospitalet, Copenhagen, Denmark. 2Cluster for Molecular Imaging, Department of Biomedical Sciences, Faculty of Health and Medical Sciences, University of Copenhagen, Denmark 3Renal, Cardiovascular, and Pulmonary Research, Department of Biomedical Sciences, Faculty of Health and Medical Sciences, University of Copenhagen, Denmark. 4Centre for Physical Activity Research, University Hospital Copenhagen – Rigshospitalet, Copenhagen, Denmark. 5Neurovascular Research Laboratory, Faculty of Life Sciences and Education, University of South Wales, UK
Copyright American Society of Nuclear Cardiology

## Slide 2
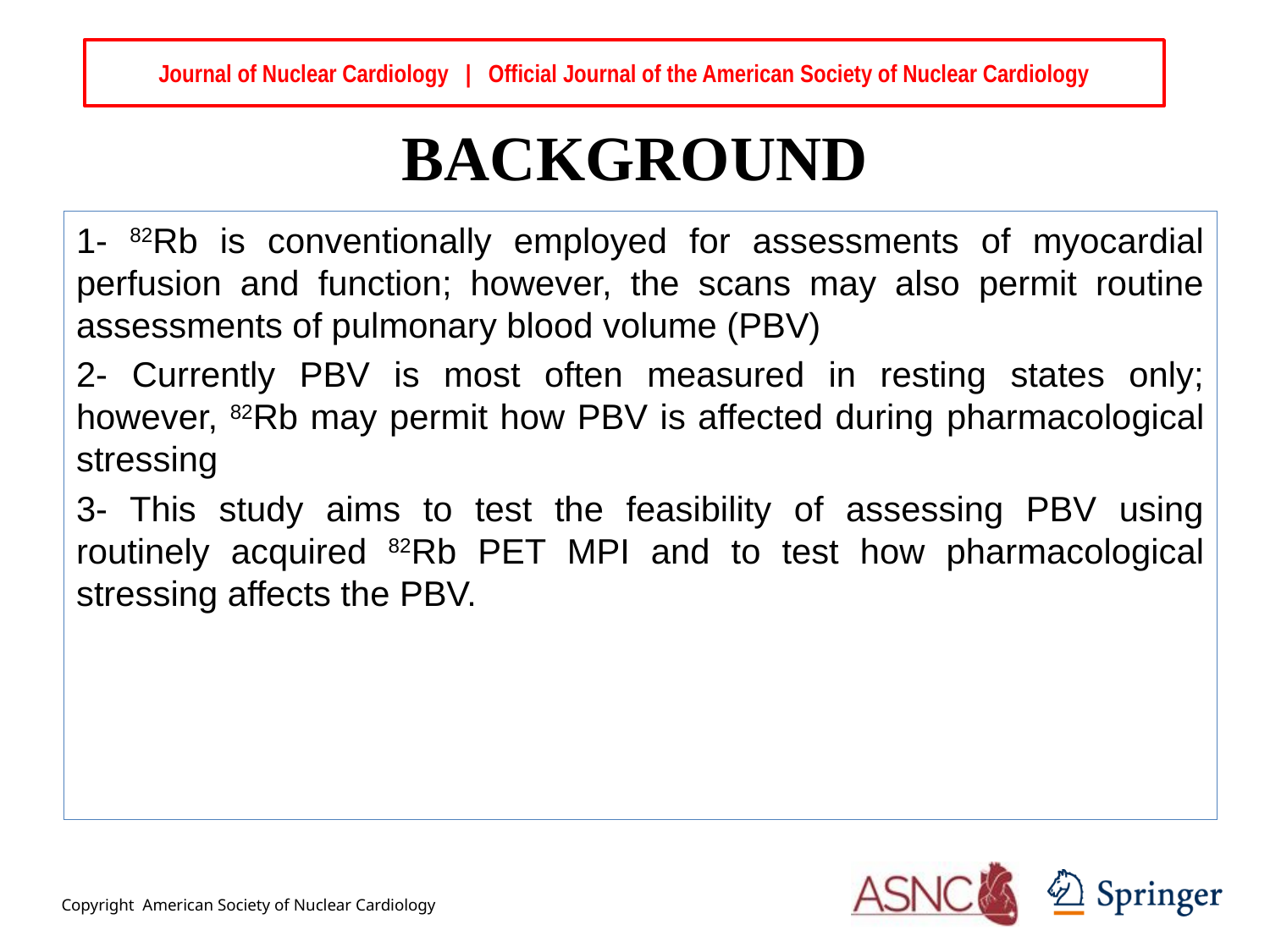

Journal of Nuclear Cardiology | Official Journal of the American Society of Nuclear Cardiology
# BACKGROUND
1- 82Rb is conventionally employed for assessments of myocardial perfusion and function; however, the scans may also permit routine assessments of pulmonary blood volume (PBV)
2- Currently PBV is most often measured in resting states only; however, 82Rb may permit how PBV is affected during pharmacological stressing
3- This study aims to test the feasibility of assessing PBV using routinely acquired 82Rb PET MPI and to test how pharmacological stressing affects the PBV.
Copyright American Society of Nuclear Cardiology

## Slide 3
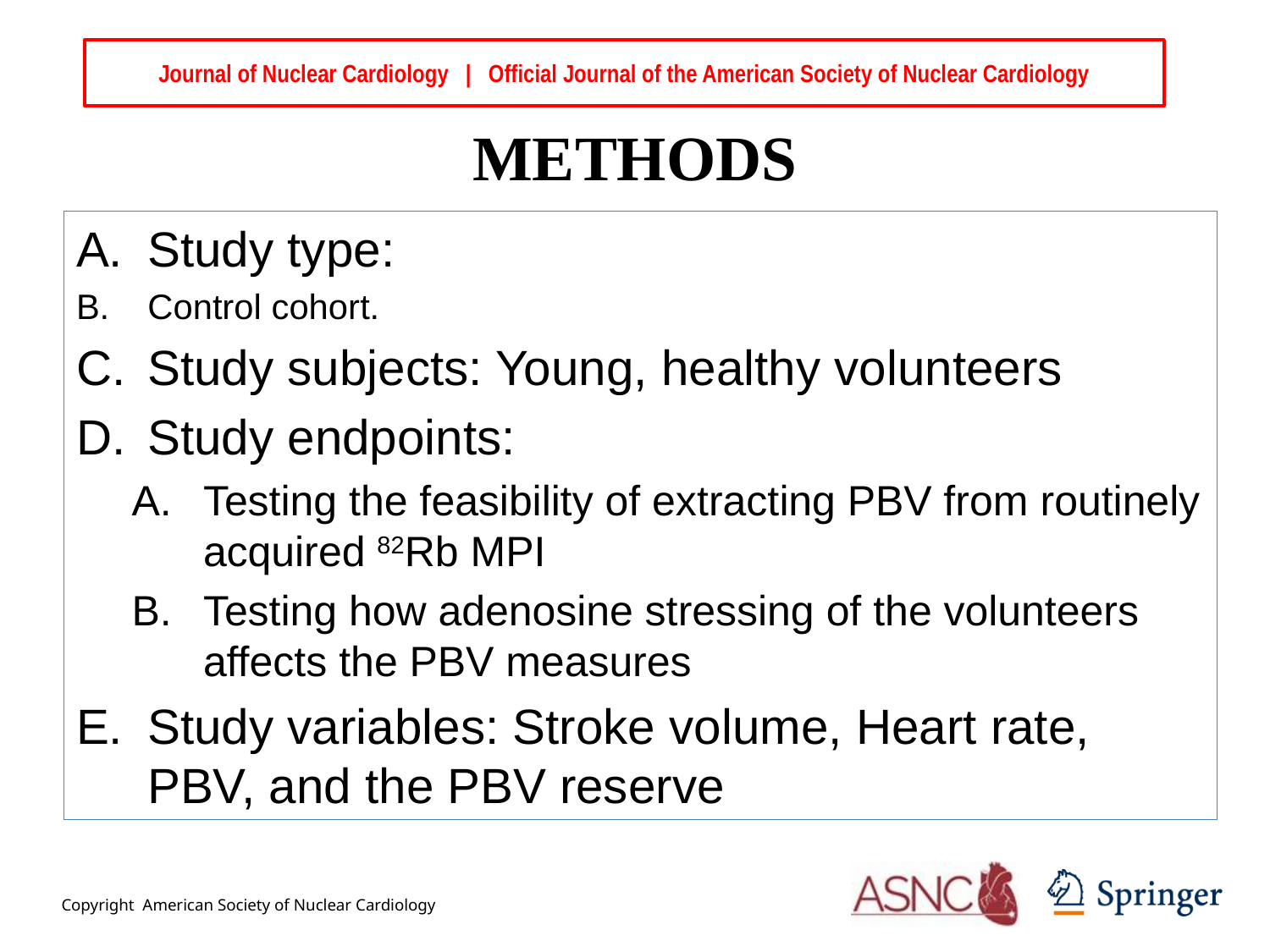

Journal of Nuclear Cardiology | Official Journal of the American Society of Nuclear Cardiology
# METHODS
Study type:
Control cohort.
Study subjects: Young, healthy volunteers
Study endpoints:
Testing the feasibility of extracting PBV from routinely acquired 82Rb MPI
Testing how adenosine stressing of the volunteers affects the PBV measures
Study variables: Stroke volume, Heart rate, PBV, and the PBV reserve
Copyright American Society of Nuclear Cardiology

## Slide 4
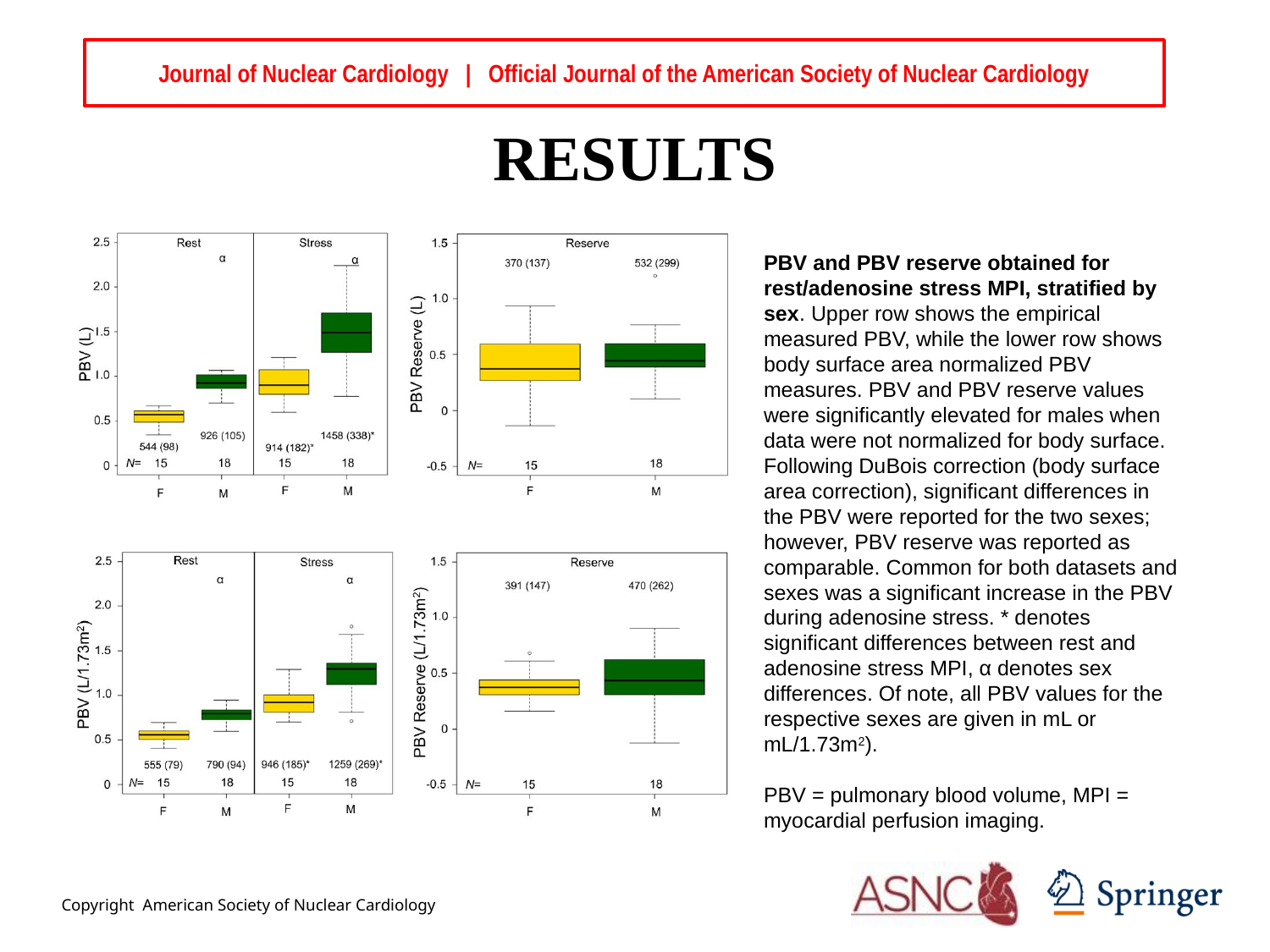

Journal of Nuclear Cardiology | Official Journal of the American Society of Nuclear Cardiology
# RESULTS
PBV and PBV reserve obtained for rest/adenosine stress MPI, stratified by sex. Upper row shows the empirical measured PBV, while the lower row shows body surface area normalized PBV measures. PBV and PBV reserve values were significantly elevated for males when data were not normalized for body surface. Following DuBois correction (body surface area correction), significant differences in the PBV were reported for the two sexes; however, PBV reserve was reported as comparable. Common for both datasets and sexes was a significant increase in the PBV during adenosine stress. * denotes significant differences between rest and adenosine stress MPI, α denotes sex differences. Of note, all PBV values for the respective sexes are given in mL or mL/1.73m2).
PBV = pulmonary blood volume, MPI = myocardial perfusion imaging.
Copyright American Society of Nuclear Cardiology

## Slide 5
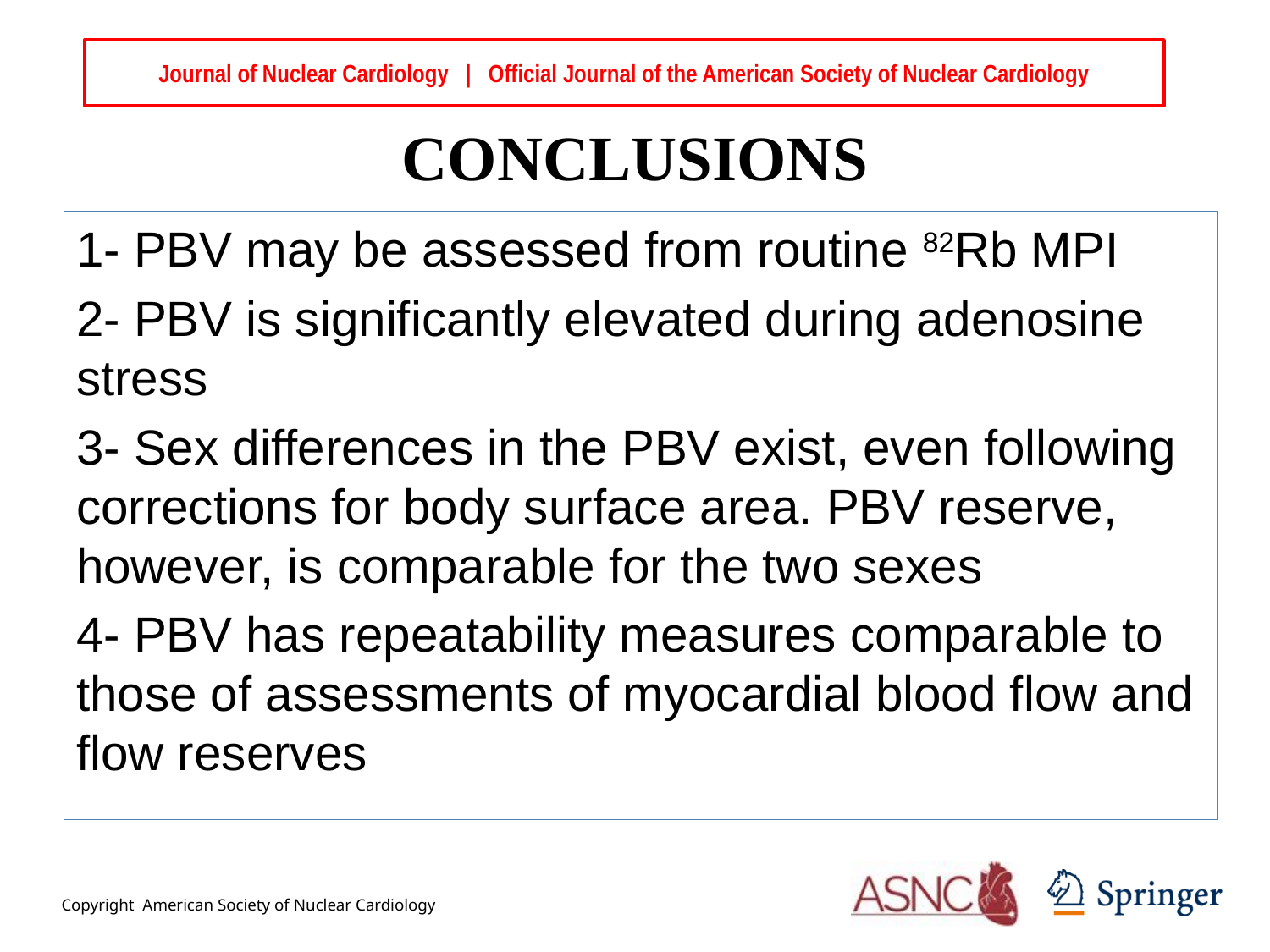

Journal of Nuclear Cardiology | Official Journal of the American Society of Nuclear Cardiology
# CONCLUSIONS
1- PBV may be assessed from routine 82Rb MPI
2- PBV is significantly elevated during adenosine stress
3- Sex differences in the PBV exist, even following corrections for body surface area. PBV reserve, however, is comparable for the two sexes
4- PBV has repeatability measures comparable to those of assessments of myocardial blood flow and flow reserves
Copyright American Society of Nuclear Cardiology
